# Difference in Pro-Inflammatory Cytokine Responses Induced in THP1 Cells by Particulate Matter Collected on Days with and without ASIAN Dust Storms

**DOI:** 10.3390/ijerph120707725

**Published:** 2015-07-09

**Authors:** Masanari Watanabe, Jun Kurai, Hiroyuki Sano, Akira Yamasaki, Eiji Shimizu

**Affiliations:** 1 Department of Respiratory Medicine and Rheumatology, Tottori University Faculty of Medicine, 36-1 Nishi-cho, Yonago 683-8504, Japan; E-Mails: junkurajun@gmail.com (J.K.); yamasaki@med.tottori-u.ac.jp (A.Y.); eiji@med.tottori-u.ac.jp (E.S.); 2 Department of Respiratory Medicine and Allergology, Kinki University Faculty of Medicine, 377-2 Ohnohigashi, Osakasayama 589-0014, Japan; E-Mail: hsano@med.kindai.ac.jp

**Keywords:** Asian dust storm, particulate matter, cytokine, THP1

## Abstract

The associations between particulate matter from Asian dust storms (ADS) and health disorders differ among studies, and the underlying mechanisms remain unclear. In this study, ADS and non-ADS particles were tested for their potential to induce pro-inflammatory cytokines associated with adverse respiratory effects. Particulate matter was collected in Japan during four periods in 2013 (2 × ADS periods; 2 × non-ADS). THP1 cells were exposed to this particulate matter, and the levels of various interleukins (ILs), and tumor necrosis factor (TNF)-α were measured. Levels of IL-2 increased significantly following exposure to all particulate matter samples (compared to levels in a solvent control). Increased levels of IL-10 and TNF-α were also observed following exposure to particles collected during three (one ADS and two non-ADS) and two (one ADS and one non-ADS) collection periods, respectively. Thus, the effects of particulate matter on cytokine responses differed according to collection period, and the effects of ADS particles differed for each ADS event. Additionally, the levels of pro-inflammatory cytokines induced by ADS particles were not always higher than those induced by non-ADS particles.

## 1. Introduction

Numerous epidemiology research studies have shown the effects of ambient pollutants on the development of health disorders [1,2,3]. Globally, ambient pollutants are now the third leading contributor to disability-adjusted life years associated with chronic respiratory disease [4]. Particulate matter is an important source of ambient pollution. It is categorized on the basis on particle size as PM_10_, PM_2.5_, or PM_0.5_, which represent median aerodynamic diameters of less than 10, 2.5, and 0.5 μm, respectively. Recent studies have demonstrated that exposure to particulate matter correlates with human health risks [5,6], and the specialized cancer agency of the World Health Organization, the International Agency for Research on Cancer, has reported an increased risk of lung cancer as levels of exposure to particulate matter increase [7].

Turbulent winds raise large quantities of dust from desert sands and are therefore a major source of particulate matter. These sand dust emissions can induce disease in humans [8] and impact the health of distant populations because they travel far from their source [9]. Deserts in China and Central Asia produce the second largest level of dust emissions worldwide (contributing approximately 20% of the global total), and these are often referred to Asian dust storms (ADS) [10]. Sand dust is typically of geological origin and consists of, for example, silicon dioxide, aluminum oxide, iron (III) oxide, calcium oxide, and magnesium oxide [11]. This sand dust is known to induce the production of pro-inflammatory cytokines [12]. Previous studies have also shown an increase in mortality and emergency treatment hospitalization for cardiovascular and respiratory diseases associated with ADS [13,14,15]. Moreover, sand dust emissions originating from China and Central Asia now also contain anthropogenic metals, chemicals, and microorganisms introduced by rapid industrial development; thus, they may pose an even greater health risk than previously thought [16,17].

Not all studies, however, have established an association between particulate matter and respiratory disorders [18,19,20,21]. Differences in the findings between studies may be attributable to the complexity of dealing with the individual variability of the studies and the disparity in the composition of particulate matter [22,23,24,25]. Several studies from Taiwan have reported no significant association between ADS and cardiovascular, cerebrovascular, and pulmonary disease [26,27,28,29,30]. Our previous study was unable to find similar effects of ADS on pulmonary function in Japanese children with and without asthma who were assessed from 2012 to 2013, despite the study being conducted with the same children [31].

Particulate matter is continuously affected by both stationary (power plants, industries, incinerators, and residential heating) and mobile sources (road traffic) [32,33,34]. It can also change size, morphology, phase states, and chemical composition via coagulation, condensation, and chemical reactions [35]. In one study, the inflammatory potential of such ambient particulate matter exhibited heterogeneity in relation to city and season [36]. Considering these factors, the various adverse respiratory effects induced by exposure to ADS may be related to the production of pro-inflammatory cytokines, and whether cytokines are produced may depend on the composition of particulate matter. To date, few studies have investigated the differences in inflammatory responses caused by particulate matter generated on days with and without ADS. The primary aim of the present study was to assess these potential differences and investigate the underlying mechanisms of these responses. Therefore, the level of pro-inflammatory cytokines induced in monocytes by exposure to ADS particles and non-ADS particles was evaluated here. Furthermore, we studied the effects of metal components in particulate matter on the production of pro-inflammatory cytokines.

## 2. Materials and Methods

### 2.1. Collection and Preparation of Airborne Particles

Airborne particles were collected in Yurihama, Tottori, using a high-volume air sampler (HV-1000R; Shibata Co., Ltd., Tokyo, Japan). The collection days were 8 to 10 March, 19 and 20 March, 8 to 15 April, and from 30 April to 6 May, all in 2013. The period of ADS exposure was determined using relevant information from the Japan Meteorological Agency originating from meteorological satellites, which was based on the criterion that visibility <10 km was due to dust arising from the deserts of East Asia. 8 to 10, 19, and 20 March were designated as ADS days; thus, particulate matter collected during these days was defined as ADS particles. Particulate matter collected during 8 to 15 April and from 30 April to 6 May was defined as non-ADS particles. Yurihama is rural and the source of air pollutants is limited to motor vehicles. Additionally, the observatory in Yurihama is not located close to populated areas. Airborne particles <10 μm were collected with an Andersen sampler (Shibata) and dried in a desiccator before and after sampling in order to facilitate the measurement of their dry weight. This dust was sterilized at 121 °C for 30 min in an autoclave (Tomy SX-300; Tomy, Tokyo, Japan) to prevent growth of bacteria and fungi, and subsequently dried at 80 °C for 4 h with a drying sterilizer (SG600; Yamato Scientific Co., Ltd., Tokyo, Japan). For stimulation, the airborne particles were diluted to 200 μg/mL in distilled deionized water.

### 2.2. Cell Line Culture

THP1 (ATCC^®^ TIB-202™) human monocyte cell lines were cultured in Roswell Park Memorial Institute medium 1640 containing 10% (*v*/*v*) fetal bovine serum, 0.05 mM 2-mercaptoethanol, 100 U/mL penicillin, 100 μg/mL streptomycin, and 0.5 μg/mL amphotericin B (Wako Pure Chemicals, Osaka, Japan) at 37 °C, 5% CO_2_, and in a humidified cell culture incubator. The pH of the particles was measured with a pH meter (MP220; Mettler Toledo, Schwerzenbach, Switzerland).

### 2.3. Cytokine Quantification

THP1 cells (1 × 10^5^ cells/100 μL/well) in 96-well plates were stimulated for 24 h with solvent only (negative control), and 200 μg/mL of the various airborne particles. Supernatants were collected and the amount of interleukin (IL)-2, IL-10, IL-12, and tumor necrosis factor (TNF)-α were measured by using the Bio-Plex Human Cytokine Th1/Th2 Assay (Bio-Rad, Hercules, CA, USA) according to the manufacturer’s protocols.

### 2.4. Measurement of Metal Elements

For airborne particles collected during all collection periods, metal elements were measured by Oki Engineering (Tokyo, Japan). The concentrations of aluminum, arsenic, barium, calcium, cadmium, chromium, copper, iron, magnesium, manganese, sodium, nickel, lead, strontium, titanium, and zinc were all measured by inductively coupled plasma atomic emission spectrometry. Silicon was measured using electrothermal atomic absorption spectrometry.

### 2.5. Statistical Analyses

Results are shown as the mean ± standard deviation (SD). SPSS statistical software (Japanese ver. 21.0 for Windows; IBM Japan, Tokyo, Japan) was used to conduct statistical analyses. One-way ANOVA followed by Turkey’s test was used to compare the effects of the various airborne particles. All quoted P values are two-sided and the significance level was set at *p* < 0.05.

## 3. Results

### 3.1. Production of Pro-Inflammatory Cytokines Caused by Airborne Particles

**Figure 1:**
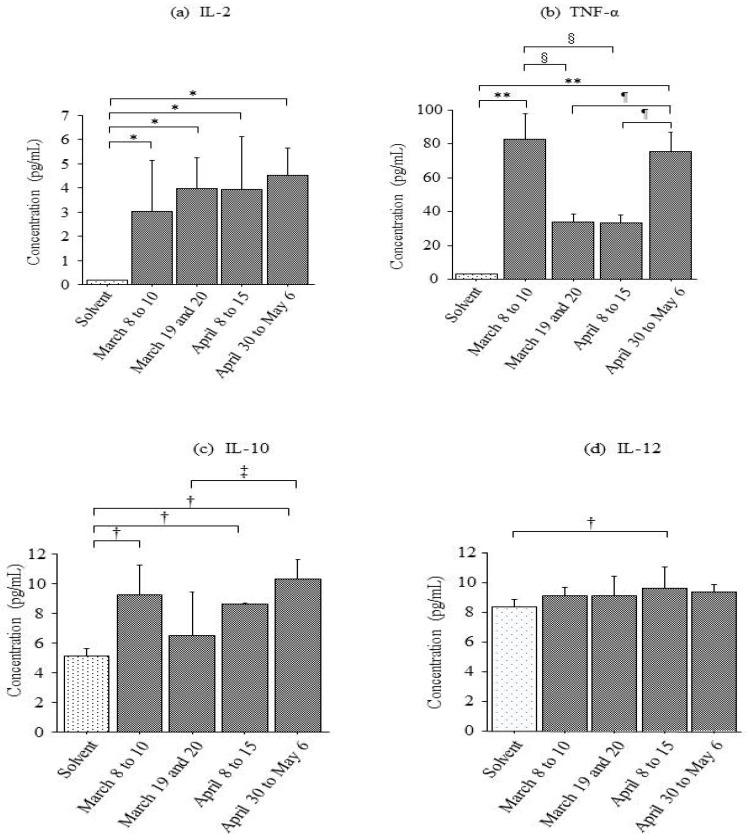
The levels of (**a**) interleukin (IL)-2; (**b**) tumor necrosis factor (TNF)-α; (**c**) IL-10; and (**d**) IL-12 were measured in a THP1 cell line using a Bio-Plex Human Cytokine Th1/Th2 Assay. Particulate matter was collected in 2013 from 8 to 10 March, on 19 and 20 March, from 8 to 15 April, and from 30 April to 6 May. Particulate matter collected from 8 to 10 March and on March 19 and 20 was defined as Asian dust storm (ADS) particles, whereas matter collected from 8 to 15 April and from 30 April to 6 May represented non-ADS particles. Cells were treated with solvent only (*n* = 4, negative control) and particulate matter (all *n* = 4; 200 μg/mL). * *p* < 0.05 *vs.* solvent, ** *p* < 0.0005 *vs*. solvent, † *p* < 0.05 *vs.* solvent, § *p* < 0.01 *vs.* particulate matter collected from 8 to 10 March, *p* < 0.05 *vs.* particulate matter collected from 30 April to 6 May, ‡ *p* < 0.03 *vs.* particulate matter collected from 30 April to 6 May.

The pH levels of the particles collected on various days were as follows: pH 7.9 on 8 to10 March (ADS), pH 8.0 on 19 and 20 March (ADS), pH 7.6 on 8 to 15 April (non-ADS), and pH 7.8 on 30 April through 6 May (non-ADS). Allowing for the simultaneous quantitative measurement of nine pro-inflammatory cytokines in a single sample, exposure of THP1 cells to airborne particles from various periods significantly induced the expression of IL-2, IL-10, IL-12, and TNF-α. There were significant differences in the increase of IL-2 caused by all airborne particles compared with levels caused by the solvent only (Figure 1a). The observed levels of TNF-α were significantly higher when cells were exposed to particles collected on March 8 to 10 (ADS) and 30 April through 6 May (non-ADS) rather than particles collected on 19 and 20 March (ADS) and 8 to15 April (non-ADS) (or the solvent control) (Figure 1b). Only ADS particulate matter collected on 19 and 20 March did not significantly increase the levels of IL-10 in cells compared with IL-10 levels produced by the solvent (Figure 1c).

Non-ADS particulate matter collected from 8 to 15 April was the only sample to cause a significant increase in IL-12 levels compared with those levels observed following solvent treatment (Figure 1d).

### 3.2. Concentration of Metal Elements

The concentrations of metal elements in particulate matter collected during each period are shown in Table 1.

**Table 1:** Concentration of metal elements (μg/mg) in collected airborne particles.

Metals	8 to 10 March	19 and 20 March	8 to 15 April	30 April to 6 May
Al	22.4	14.8	23.8	27.4
As	ND	ND	ND	ND
Ba	0.18	0.10	0.16	0.11
Ca	44.0	31.2	44.0	28.1
Cd	ND	ND	ND	ND
Cr	ND	ND	ND	ND
Cu	ND	0.07	0.11	0.13
Fe	22.3	16.8	15.4	21.5
Mg	16.8	14.4	16.0	13.7
Mn	0.56	0.40	0.48	0.49
Ni	0.15	0.11	0.12	0.13
Pb	0.12	0.06	0.08	0.06
Si	108.0	72.0	116.0	133.0
Sr	0.22	0.16	0.16	0.15
Ti	0.92	0.52	0.76	0.91
Zn	0.64	0.60	0.48	0.49

Particulate matter collected from 8 to 10 March and on 19 and 20 March was defined as Asian dust storm (ADS) particles, whereas matter collected from 8 to 15 April and from 30 April to 6 May was defined as non-ADS particles. ND: Not detected.

## 4. Discussion

In this study, we collected particulate matter which was <10 μm in diameter in Western Japan, across four periods from March to May 2013, with two periods being classed as ADS days and two being classed as non-ADS days. The effects of these particulate matters on the production of various pro-inflammatory cytokines in monocytes were then assessed according to the period in which the particles were collected. Our key finding was that particulate matter induced significant production of the pro-inflammatory cytokines IL-2, IL-10, IL-12, and TNF-α from monocytes. The effects of particulate matter on the production of pro-inflammatory cytokines differed according to the period of collection. For example, the effects of ADS particles on cytokine responses were different for each of the ADS periods (*i.e.*, 8 to 10 March *vs.* 19 and 20 March). Furthermore, particulate matter collected during ADS days did not always have a higher potency for production of pro-inflammatory cytokines when compared with that collected on non-ADS days.

Many epidemiological studies have attempted to investigate the effects of short-term exposure to particulate matter on pulmonary function and respiratory symptoms, but several investigations have been unsuccessful [18,19,20,21]. Similarly, previous epidemiological studies have demonstrated heterogeneities in the health disorders linked to exposure to sand dust emission in East Asia. Based on analysis of its morphological, chemical, physical, and thermodynamic properties, a large proportion of fine and ultrafine particulate matter has anthropogenic origins, e.g., emissions from combustion or motor vehicles [37]. In addition, ambient coarse particulate matter and sand dust also include components of geological origin [11,37]. Acute effects triggered by short-term exposure to such particles can exacerbate inflammatory responses, and this has implications for health because inflammation is associated with the long-term development of lung disease and possibly particle-induced cardiovascular disease [38,39,40]. Dusts of geological origin are known to induce the production of pro-inflammatory cytokines [12]. Additionally, Kumar *et al.* [41] showed that ambient particulate matter was more important than traffic-derived particulate matter in causing injury to airway epithelial cells leading to production of pro-inflammatory cytokines. Experimental studies have also repeatedly found that the coarse fraction is the most injurious to cells, even when other size fractions are delivered at the same mass/concentration [36,42]. However, several studies have reported contrasting findings, specifically that the associations between chemical compositions and particle toxicity tended to be stronger for fine and ultrafine particulate matter [43,44]. Collectively, these findings show that the effects of particulate matter on the production of pro-inflammatory cytokines can vary considerably, and that the composition of particulate matter affects the observed differences. In our study, it is possible that the composition of particulate matter caused the observed differences in the production of pro-inflammatory cytokines. According to our analysis, particulate matter collected during sand dust emissions in East Asia varies in its inflammatory potential.

Kumar *et al.* [41] compared the production of pro-inflammatory cytokines among various sources of particulate matter and suggested that the iron content of airborne particulate matter might be important in airway epithelial injury. A recent *in vivo* study has also demonstrated an association between the iron content of inhaled particulates and deficits in pulmonary function [44]. In the present study, we did not detect a statistical difference between the concentrations of metal elements between particulate matter collected on ADS *vs.* non-ADS days. Similarly, the effect of these particles on THP-1 cytokine production did not differ significantly. Some previous studies have suggested that certain metals such as iron have a role as redox-active metals in triggering the secretion of inflammatory mediators by cells exposed to particulate pollutants [45,46,47,48]. Measuring oxidase stress in cells may help to determine which metal components play an important role in the production of pro-inflammatory cytokines. Such analyses were not possible here because we used nearly the majority of particulate matter to obtain the present results. However, in future research, it would be useful to measure the oxidase stress induced by cells after stimulation with particulate matter.

Other components of particulate matter may also have the potential to induce the production of pro-inflammatory cytokines. Previous studies have shown that oxidative stress and increased production of pro-inflammatory cytokines were related to the organic compounds associated with particulate matter [49,50]. Other relevant biological materials that might be associated with the particulates include microorganisms, fungal spores, and pollens [42]. In studies that used macrophages, cellular injury was associated with the endotoxin content of particulate matter [36,51]. We have previously found that endotoxins in airborne particles collected during ADS days augmented, to a small degree, the production of pro-inflammatory cytokines in monocytes [52].

Heavy dust that originates from deserts in East Asia depending on the atmospheric transport route can be classified into three types: Dust from Type 1 events with high counts of aerosolized air pollutants, dust from Type 2 events with high counts of mineral dust particles relative to aerosolized air pollutant counts, and dust from Type 3 events with very low counts of aerosolized air pollutants [53]. In the present study, the inflammatory potential of particulate matter from ADS varied. A previous study showed that coarse particulate matter collected in Australia during a period that included a dust storm did not increase production of pro-inflammatory cytokines in comparison to other ambient particulate matter [41]. There is considerable evidence to suggest that dusts of geological origin can cause significant respiratory inflammation [12], but various anthropogenic substances attached to sand dust may also affect differences in the inflammatory potential of airborne ADS particulate matter. Further study is therefore necessary to test whether dusts of geological origin and anthropogenic substances show strong differences in their potential for induction of inflammatory responses.

This study had several limitations, which are as follows: first, we were unable to estimate all pro-inflammatory cytokines, that monocytes have the potential to produce, because the Bio-Plex Human Cytokine Th1/Th2 Assay can only measure certain cytokines. TNF-α and IL-6 are the cytokines most often selected to investigate the effects of particulate matter on *in vitro* inflammatory responses [54]. When we used the same particulate matter in a preliminary experiment to investigate the production of IL-6 using an enzyme-linked immunosorbent assay, the result was similar to that for TNF-α. Second, we were unable to measure the distribution of PM_2.5_ in the collected airborne particles. The effects of particulate matter on the production of pro-inflammatory cytokines may depend on their size. Further research should therefore be conducted to measure the configuration ratio between PM_2.5_ and PM_10_ in ADS *vs.* non-ADS particles. Third, only four sampling periods, including collection during two ADS periods and two non-ADS periods, were used in this study. Although the number of sampling periods is low, our previous studies suggested that the potential to induce pro-inflammatory cytokines was significantly associated with pulmonary function and respiratory symptoms in adult patients with asthma and schoolchildren [31,52]. Therefore, we believe it is reasonable to conclude that the effects of particulate matter on cytokine responses differed according to collection period, and that the effects of ADS particles differed for each ADS event. Fourth, we could not fully analyze the composition of particulate matter for biological materials, chemicals, and organic compounds because a sufficient amount could not be collected. Finally, for the same reasons, we could not consider effects on other cell lines. Many studies have chosen to test airway epithelial cells as well as monocytes/macrophages because they are strongly exposed to particulate matter in the environment. It may be most useful to test primary human cells; however, this might be difficult due to their limited availability and life span.

## 5. Conclusions

Given the stated limitations, the results of this study indicate that the production of pro-inflammatory cytokines differs depending on the composition of particulate matter. Particulate matter collected during ADS days did not always have a higher capacity for induction of pro-inflammatory cytokines than that collected on non-ADS days. Additionally, the effects of ADS airborne particles on inflammatory responses differed with each ADS event.

## Abbreviations

ADS	Asian dust storm	IL	interleukin
TNF	tumor necrosis factor	PM_10_	particulate matter smaller than 10 μm
PM_2.5_	particulate matter smaller than 2.5μm	PM_0.5_	particulate matter smaller than 0.5μm
SD	standard deviation		
